# Unilateral isolated sphenoid sinusitis with contralateral abducens nerve palsy - A rare complication treated in a low-resource setting

**DOI:** 10.1186/s40463-015-0053-y

**Published:** 2015-03-01

**Authors:** Jennifer Siu, Sudhir Sharma, Leigh Sowerby

**Affiliations:** School of Medicine, Queen’s University, Kingston, ON Canada; Department of Otolaryngology – Head and Neck Surgery, Georgetown Public Hospital, Georgetown, Guyana; Department of Otolaryngology – Head and Neck Surgery, Schulich School of Medicine, University of Western Ontario, London, ON Canada

**Keywords:** ISSD (Isolated Sphenoid Sinus Disease), Abducens nerve palsy, Transnasal sphenoidotomy, Paranasal sinuses

## Abstract

**Background:**

Unilateral isolated sphenoid sinus disease (ISSD) is a rare diagnosis of the paranasal sinuses that can be associated with complications involving vascular, neurologic, and optic structures in close proximity.

**Case presentation:**

A 62 year old female presented to a hospital in Georgetown, Guyana with right lateral rectus palsy, diplopia, and a severe progressively worsening headache. CT scan revealed an opaque left sphenoid sinus consistent with unilateral ISSD. A transnasal sphenoidotomy was performed without complication under local anesthetic in the absence of endoscopic guidance. The patient's headache resolved immediately after surgery while the diplopia and lateral rectus palsy resolved completely after 6 weeks.

**Conclusion:**

We present a rare complication of ISSD and highlight challenges associated with diagnosis and management of ISSD in a resource-limited setting. This is the second reported case of unilateral ISSD with contralateral lateral rectus palsy in the literature.

## Background

Guyana, a former British colony with a population of 751,000 is one of the poorest countries of the Americas and has one of the lowest health status indicators of its region [1,2]. There are less than 50 physicians per 100,000 and only a handful of Otolaryngology specialists [3]. Georgetown Public Hospital Corporation (GPHC) serves as the national tertiary care teaching hospital where the majority of complicated otolaryngology cases are seen.

Otolaryngologists at GPHC are faced daily with challenges of providing care despite a lack of modern surgical equipment such as operating microscopes, and instruments for functional endoscopic sinus surgery and otologic surgery. Other challenges include a lack of consumables, insufficient operating room staff, insufficient operating room time, an outdated academic library, poor maintenance of equipment, and a lack of a post graduate program in Otolaryngology. It is within this context that we describe a rare case of unilateral isolated sphenoid sinus disease (ISSD) with contralateral lateral abducens nerve palsy.

ISSD is a rare disease which accounts for 1–2.7% of all paranasal sinus pathology [4-7]. ISSD can be difficult to diagnose due to its subtle onset and typical presentation of non-specific facial pain or headache [8]. If the disease process is severe, ISSD can disrupt important nearby structures including the pituitary gland, cavernous sinus, internal carotid, and several cranial nerves [9]. Cranial nerve involvement is a rare complication of ISSD and abducens nerve palsy is particularly uncommon, with only a handful of documented abducens nerve palsies, most often presenting bilateral or ipsilateral to the side of the ISSD.

We describe a patient with unilateral ISSD complicated by contralateral abducens nerve palsy. The patient was successfully treated with medical therapy and a sphenoidotomy under local anesthetic without endoscopic guidance. This is the second case of unilateral ISSD with contralateral abducens nerve palsy reported in the literature [10].

## Case presentation

A sixty-two year-old female presented to the Emergency Room at Georgetown Public Hospital Corporation, Georgetown, Guyana, with complaints of diplopia accompanied by a progressively worsening headache localized to the left lateral aspect of her head. There was no present or past history of cough, cold or sinus problem, fever, or vomiting. The patient was otherwise healthy with no significant past medical or surgical history.

**Figure 1:**
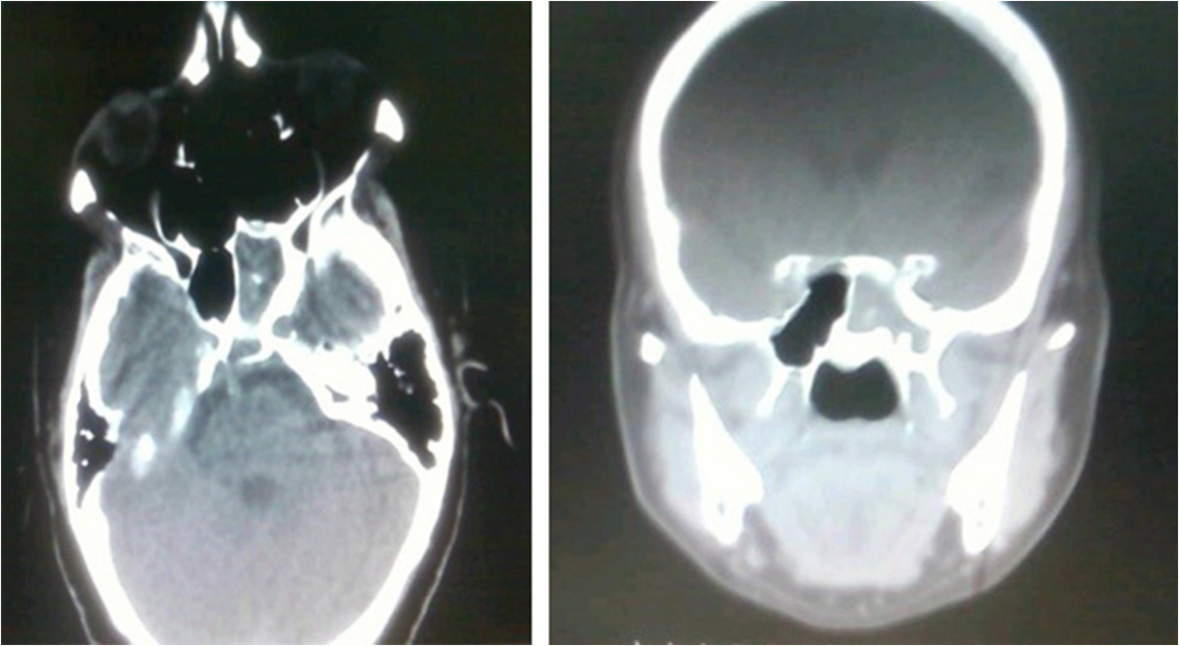
**Patient’s CT scan of the paranasal sinuses.** Opacification in the left sphenoid sinus with hypertrophic mucosa and secretions. Left: Axial view. Right: Coronal view.

A complete head and neck examination was unremarkable aside from a lateral rectus palsy present in the right eye. Computerized tomography scanning (CT) of the paranasal sinuses revealed opacification with hypertrophic mucosa and secretions in the left sphenoid sinus (Figure 1).

A course of broad spectrum antibiotics (amoxicillin-clavulanate 125 mg PO BID and gentamicin 80 mg IM BID) was administered in addition to nasal decongestants, and analgesics. The patient’s symptoms persisted despite 48 hours of medical therapy. The decision was made to perform a trans-nasal sphenoidotomy in order to avoid progression and complications of the disease.

The surgery was performed with the patient in semi-recumbent position under local anesthesia with diazepam 2 mg IV used for mild sedation. The right nostril was sprayed with 10% xylocaine. A nasal speculum and headlight were used for the procedure as functional endoscopic equipment was not available. The middle turbinate was displaced laterally and the inferior aspect of the superior turbinate was removed. A probe was used to identify and landmark the anterior wall of the sphenoid sinus and ostium. A trocar and cannula were used to open the ostium and straight punch forceps were used to widen the anterior sphenoidal wall. Immediate aspiration revealed purulent discharge which was sent for culture and sensitivity. The sinus was irrigated through the cannula with normal saline with the patient sitting up and bent forward. The patient tolerated the procedure well and there were no perioperative complications. The microbiology report showed *Streptococcus pneumonia* and *Staphylococcus aureus,* consistent with respiratory flora. The same course of preoperative antibiotics were continued during the hospital stay and the patient was given a 2 week course of amoxicillin-clavulanate 625 mg PO BID at discharge.

**Figure 2:**
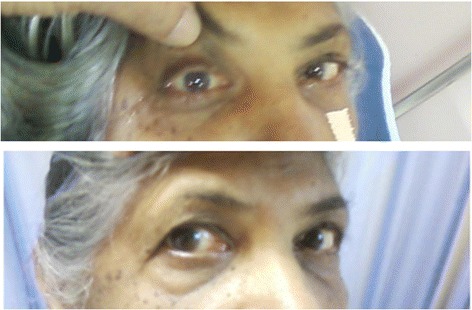
**Examination of extraocular movements.** Above: Right lateral rectus palsy prior to sphenoidotomy. Below: Full recovery of abduction in right eye 6 weeks following sphenoidotomy.

At the 6 week follow-up appointment, the patient was asymptomatic with complete resolution of the lateral rectus palsy, headache, and diplopia (Figure 2). No disease sequelae were detected at the subsequent 6 month follow up visit.

## Discussion

ISSD, or isolated sphenoid sinus disease is a rare pathology that accounts for 1–2.7% of all sinus disease [4-7]. ISSD is associated with several diagnostic and therapeutic challenges due to its ambiguous presentation, potential involvement of other related structures, and difficult anatomic access for surgical intervention. This case is presented within the context of a healthcare centre in Guyana, one of the poorest countries in the Americas, where the challenges of ISSD management are compounded with the limitations of providing medical service in a low-resource setting.

**Figure 3:**
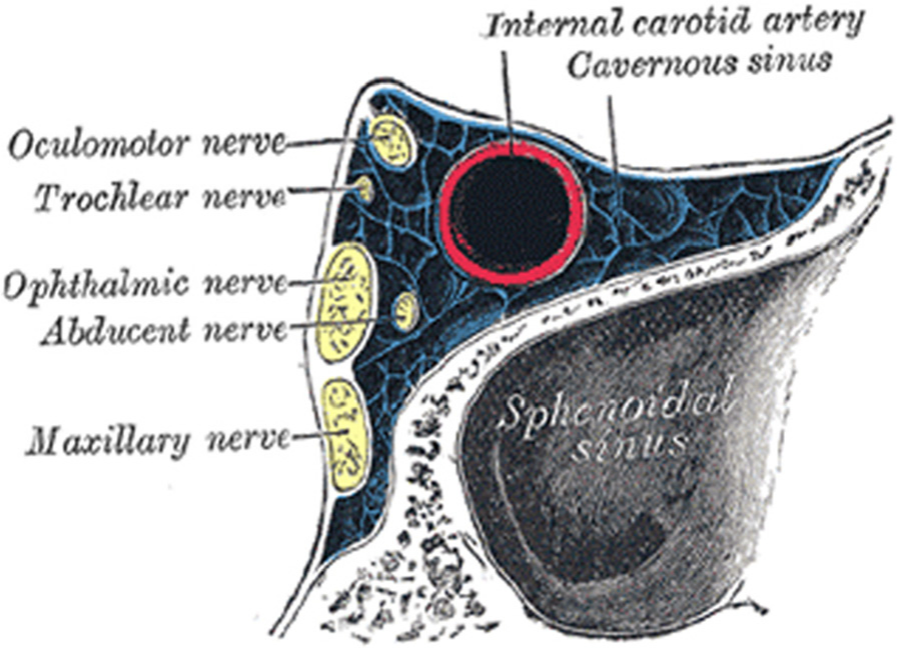
**Coronal section of the posterior aspect of the sphenoid sinus showing its close proximity to important functional structures: the internal carotid artery, cavernous sinus, and oculomotor, trochlear, ophthalmic, abducens, and maxillary nerves [** 13 **].**

Prompt diagnosis and surgical intervention of ISSD is crucial in order to prevent serious complications caused by disease spread to the intracranial and orbital regions and involvement of important structures including the cranial nerves, pituitary gland, cavernous sinus, and internal carotid artery (Figure 3) [9]. Previous reports demonstrate that a delayed diagnosis of ISSD has been shown to result in increased morbidity with 23-29% of cases left with permanent disability [11,12].

However, diagnosis of ISSD may be difficult due to its insidious onset and common presentation of vague facial pain or headache. Less often, patients present with a variety of different symptoms associated with cranial nerve involvement including blindness or visual loss (CN2), ptosis (CN3), retro-orbital pain and mid-facial pain/numbness (CN5), or diplopia (CN6) [14,15].

In this case report, the patient presented with a severe headache and lateral rectus palsy. Involvement of the abducens nerve (CN6) is uncommon, with only a few documented cases of ISSD with ipsilateral or bilateral abducens nerve palsy [16-18], and only one case of ISSD with contralateral palsy [10].

Nasal endoscopy is an important component of the examination of patients with ISSD in order inspect the sphenoethmoidal recess, identify anatomic landscapes for preoperative planning, and to obtain material for culture [19]. However, in healthcare centres where endoscopes are not available, such direct visualization and magnification is not possible. The use of a nasal speculum and headlight alone significantly limits the extent of evaluation of the nasal mucosa, sinonasal anatomy and nasal pathology prior to surgery.

CT imaging is the gold standard for diagnosis of ISSD. The scans can delineate the extent of the disease, and relationship to the optic nerves and internal carotid arteries. Results from the scan can also help categorize the etiology of the disease as either inflammatory (sinusitis or mycetoma) or non-inflammatory (CSF leak or mucocele), depending on the extent of air-fluid levels, mucosal thickening, expansion of sinus walls, opacification, chronic osteitis, and bony dehiscence [7,19]. MRI can provide additional information on whether the disease is neoplastic in nature or has a vascular origin such as cavernous hemangioma, internal carotid artery aneurysm, carotid-cavernous fistula, or hemangiopericytoma [19].

Medical imaging presents the second challenge to managing sinus disease in Guyana. Although GPHC is a public healthcare facility where most medical treatments and surgical procedures are government funded, advanced medical imaging such as CT and MRI are exclusively performed in separate private health care centres. Patients are required to pay out of pocket for these procedures which cost approximately $150USD and $400USD respectively. In a country where the gross national income per capita is approximately $3500USD, many patients cannot afford what would be considered the requisite medical imaging.

Fortunately, the patient in this case was able to pay for a CT scan which showed opacification in the left sphenoid sinus with hypertrophic mucosa and secretions, consistent with acute or chronic sinusitis, a mucous retention cyst, polyp or mucocele [7,19]. An MRI would have been ideal to rule out cavernous sinus involvement as the etiology for the contralateral abducens nerve palsy, however this was not financially possible for the patient.

Without additional imaging available for this case, it was not possible to attribute any structural cause of the contralateral abducens nerve palsy. It is possible that contralateral nerve involvement may have been due to transmission of the disease through the superior or inferior intercavernous sinus. However, even with additional imaging using MRI, the authors of a similar case report of unilateral ISSD and contralateral abducens nerve palsy could not find any objective explanation for the the contralateral symptoms. The MRI in their case did not show enhancement of the contralateral abducens or any contralateral sinus pathology. They attributed the neuropathy to the presence of an infected dominant sphenoid sinus crossing the midline, extending into the cavernous sinus and involving the contralateral structures [10].

A variety of surgical approaches can be used to treat ISSD with the goal of identifying the sphenoid ostium, restoring natural drainage of the sinus, and obtaining material for culture. Traditional approaches include the transseptal, transsphenoidal, transantral, and intranasal approaches [18]. However, endoscopic approaches are most commonly employed today, due to their superior visualization of disease process and anatomy. With the endoscopic transnasal approach, the sphenoid ostium is identified and enlarged, and endoscope is passed to evaluate the location of the carotid artery and optic nerve before enlarging the ostium to 5-10 mm in order to reduce the possibility of recurrence. Typically performed under general anesthetic, endoscopic approaches are associated with reduced operating time, decreased blood loss, and decreased morbidity compared to non-endoscopic approaches [20-22].

Although there are obvious advantages with the endoscopic approach, this specialized equipment is not available in Guyana. Furthermore, due to insufficient OR staff and OR time, general anesthesia is typically reserved for more complicated cases. Despite these limitations, the procedure was performed without complication, under sedation, with local anesthetic, and in absence of endoscopic equipment.

## Conclusions

ISSD is a rare disease that is presents several diagnostic and therapeutic challenges. Prompt diagnosis and intervention is necessary to prevent devastating complications involving important vascular, neurologic, and optic structures. Advancements in technology including CT, MRI, and endoscopic sinus surgery have allowed better elucidation of the disease process, etiology, and surgical intervention. However, otolaryngologists in low-resource countries including Guyana, must adapt to the limited resources available to diagnose and treat complicated sinus disease.

This case not only represents the second documented case of unilateral ISSD complicated by contralateral lateral rectus palsy, but also illustrates the importance of global collaborations that encourage our international colleagues to engage in the academic community and training of future generations of healthcare practitioners.

## Consent

Written informed consent was obtained from the patient for publication of this case report and any accompanying images.
